# Meridional binocular rivalry reveals a trace of uncorrected oblique input during development in the adult brain

**DOI:** 10.1038/s41598-023-35814-0

**Published:** 2023-06-19

**Authors:** Gad Serero, Maria Lev, Uri Polat

**Affiliations:** School of Optometry and Vision Science, Mina and Everard Goodman, Faculty of Life Sciences, Bar-Ilan University, Ramat Gan, Israel

**Keywords:** Neuroscience, Visual system, Object vision

## Abstract

Binocular rivalry (BR) is a visual perception phenomenon that occurs when each eye perceives different images and stimuli, causing alternating monocular dominance. To measure BR, many studies have used two monocular conflicting images to induce monocular alternations. Here we chose a group of participants with oblique astigmatism (OA) and who produced blur on the orthogonal oblique meridian in each eye, resulting in two conflicting images, which may enhance the stimulation of monocular alternations. Our results show that OA participants tend to have a high rate of BR when viewing natural images, whereas the control group does not have BR for the same images. We suggest that this low ability to fuse could indicate the presence of a trace due to uncorrected vision during the critical period, which could be retained in the adult brain.

## Introduction

Binocular rivalry (BR) is a visual perception phenomenon that occurs when different images or stimuli are perceived by each eye, presenting conflicting images at the binocular level; this prevents fusion, thus producing alternating periods of monocular dominance; perception of the other eye is suppressed^1^. BR can be affected by time and contrast^2^. These parameters have a crucial effect on the level of rivalry and, more precisely, its completeness. In fact, it has been demonstrated that high-contrast targets alternate more rapidly than low-contrast targets, which frequently blend into a composite percept^2^. Moreover, it was found that the completeness of rivalry^3^ increases together with the contrast level^2^ and the background motion^4^. In addition, it increases the time of the trial up to about 40 s^5^, and it also depends on the spatial frequency. Under dichoptic conditions, BR is manifested when the stimuli are presented for longer than 200 ms^6^.

In the last few decades, various studies on BR have led to several models (for a review, see Blake^1^). Even though stereopsis and BR seem incompatible, according to Wolfe’s^7^ model of binocular single vision, BR cohabits two independent and parallel pathways that interact by examining the visual inputs simultaneously. Most models of rivalry suggest that one set of neurons maintains dominance only temporarily until it can no longer inhibit the activity of the competing neurons, leading to a reversal in perceptual dominance. According to the hybrid model of rivalry, monocular and binocular neurons respond simultaneously to inhibitory interactions^8^. When dichoptic orthogonal patterns rival one another at the same time, this results in strong inhibition between eye-selective or pattern-selective neurons, which can alter the balance in the relative strengths of responses to the two stimuli, consequently initiating rivalry^8^. It has been shown that when neurons represent two adjacent regions of visual space, each group of neurons receives inputs from *both* eyes. Among the monocular neurons, reciprocal excitatory connections can promote grouping by the eye or interocular grouping between neurons with similar orientation preferences. Excitatory connections between binocular neurons can also lead to pattern-based grouping across adjacent regions^8^.

Astigmatism is a common refractive error. Usually, it is due to a deviation from the spherical curvature of the cornea, resulting in a distorted image along the astigmatic axis. It occurs when rays propagating in perpendicular planes through the eye focus on different distances^9^. Thus, the refractive power differs in various meridians, and consequently, there is a meridian with a high refractive error and an orthogonal meridian with a weaker refractive error. Therefore, two images will be on the retina. The classification of corneal astigmatism depends on its axis orientation. Astigmatism can be produced in two different forms, cardinal or oblique. The cardinal form of astigmatism is characterized by two different corneal curvatures: the vertical meridian has a corneal curvature that differs from the horizontal meridian. Studies^10^ show that most neonates are hyperopic with a high degree of astigmatism; the horizontal focal line obtained by the vertical corneal meridian is more blurred. After the age of 4 years, the amount of astigmatism is significantly reduced. *Oblique* astigmatism (OA) is characterized by an orientation axis between 16° and 74° or 106° and 164°; the cornea is more curved along one of the oblique axes, and it is less curved along the orthogonal, oblique axis. Consequently, the image will be blurred along the oblique meridian. Development of OA in infants is still not well understood. Owing to the rarity of oblique astigmatism, there is not enough information in the literature regarding the development and its origin. It was suggested that during the development period, the amount of astigmatism is reduced but the meridian orientation remains unchanged^11^.

In our recent study^12^ we showed that participants with OA have impaired lateral interactions in the blurred meridians of each eye, which is reminiscent of the impaired lateral interactions in meridional amblyopia^13^. Moreover, previous research suggests that visual experiences during the sensitivity period can affect the development of BR and potentially have long-term effects on visual perception^14^. Asymmetric optical inputs such as astigmatism, explained above, do not disappear without leaving traces, particularly regarding the mechanism that groups contour elements^15^. One study showed that adaptation to astigmatic blur is transferred to long-term memory that could be engaged when blur is reapplied or disengaged when it is removed^15^. This led us to suggest in a recently submitted study that optically corrected astigmatic adults exhibit a clear directional bias in shape perception (grouping) compared with non-astigmatic ones (under review: Serero, Lev, Sagi, and Polat “Traces of early development bias in adult brain”. *Scientific Reports*). This bias may represent a trace of persisting adaptive correction, developed to compensate for the biased visual input during early life before the optical correction was applied, which is only partially eliminated after it is corrected. In addition, lateral interaction can be defined as the capacity of a neuron to interact with its neighbor, and it matures by the age of 5–6 years^16^. Since normal development of visual processing is experience dependent, distorted visual input during this period may compromise normal development.

One study^13^ examined what occurs when lateral interactions differ abnormally at the axis of higher optical error (cylindrical), and it analyzed lateral interactions as a function of the higher and the lower optical blur axes in humans. Lateral interactions along the meridian with the lower refractive error (the smallest optical blur) and the meridian with the higher cylindrical error (the higher optical blur) were measured and compared. They indicate that spatial interactions might be normal in the direction of the lower refractive error, whereas facilitation is poor along the orthogonal axis, where the refractive error is highest. Thus, the process of adjusting to persistently distorted input during development may have a long-term abnormal effect on neuronal connectivity. In our recent study, we showed that participants with OA have lower collinear facilitation in the blurred meridians of each eye^12^. We suggested that these results are reminiscent of the development of meridional amblyopia and that the monocular anisotropy could be a cortical trace of uncorrected astigmatism during the development period , which is observed in adulthood^12^.

In OA, the image is blurred along the oblique meridians. Therefore, studies have shown that in most cases of OA the meridians of astigmatism in both eyes show a mirror symmetry^17^, producing two conflicting images, which may pose a problem when the monocular inputs are combined. This effect may lead to competition for monocular dominance between the eyes, consequently resulting in bi-stable natural perception and an unconscious binocular meridional rivalry. In most studies, different artificial stimuli are exposed to each eye separately, which causes different alternations between the eyes and BR. Here we hypothesized that due to astigmatic blur, BR is stimulated by naturally conflicting images. This effect may suggest that BR could indicate that an adult brain with abnormal visual processing development is due to an uncorrected OA during the early period of development.

## Results

A detailed refractive information is provided in the Methods and Table 1.

**Table 1:** Refractive status of the participants.

Participant	Right eye	Left eye	Sex
1	− 1.5	− 1.25/ − 0.75X60	F
2	pl/ − 2.25X30	pl/ − 2.25X140	M
3	− 4.75/0.75X130	− 4.00/ − 1.75X25	F
4	− 0.25/ − 1.00X120	− 0.75/0.75X40	F
5	− 1.25/ − 0.75X155	− 1.25/ − 1.00X30	M
6	− 5.50/ − 1.25X150	− 4.75/ − 0.75X60	F
7	− 4.75/ − 0.75X130	− 4.25/ − 1.75X25	F
8	− 4.00/ − 0.75X50	− 2.75/ − 1.00X130	F
9	− 1.00/ − 0.75X135	− 1.00/ − 0.75X45	M
10	− 0.75/ − 0.75X120	− 0.75/ − 0.75X65	M
11	− 2.25/ − 1.00X120	− 3.00/ − 1.00X50	M
12	− 3.25/ − 0.75X60	− 3.00/ − 0.75X150	F
13	− 0.50/1.25X150	− 0.50/1.25X35	F
14	− 1	− 1	M
15	− 2	− 1	F
16	− 4	− 4	F
17	− 0.25	− 0.25	F
18	− 0.5	− 0.75	F
19	− 1.25	− 1.25	F
20	− 1	− 1	F
21	− 0.5	− 0.75	F
22	− 0.25	− 0.25	M
23	− 0.25	− 0.25	F
24	− 4	− 4	M
25	− 0.25	− 0.5	F
26	− 2	− 2.25	F

### Experiment 1

**Figure 1:**
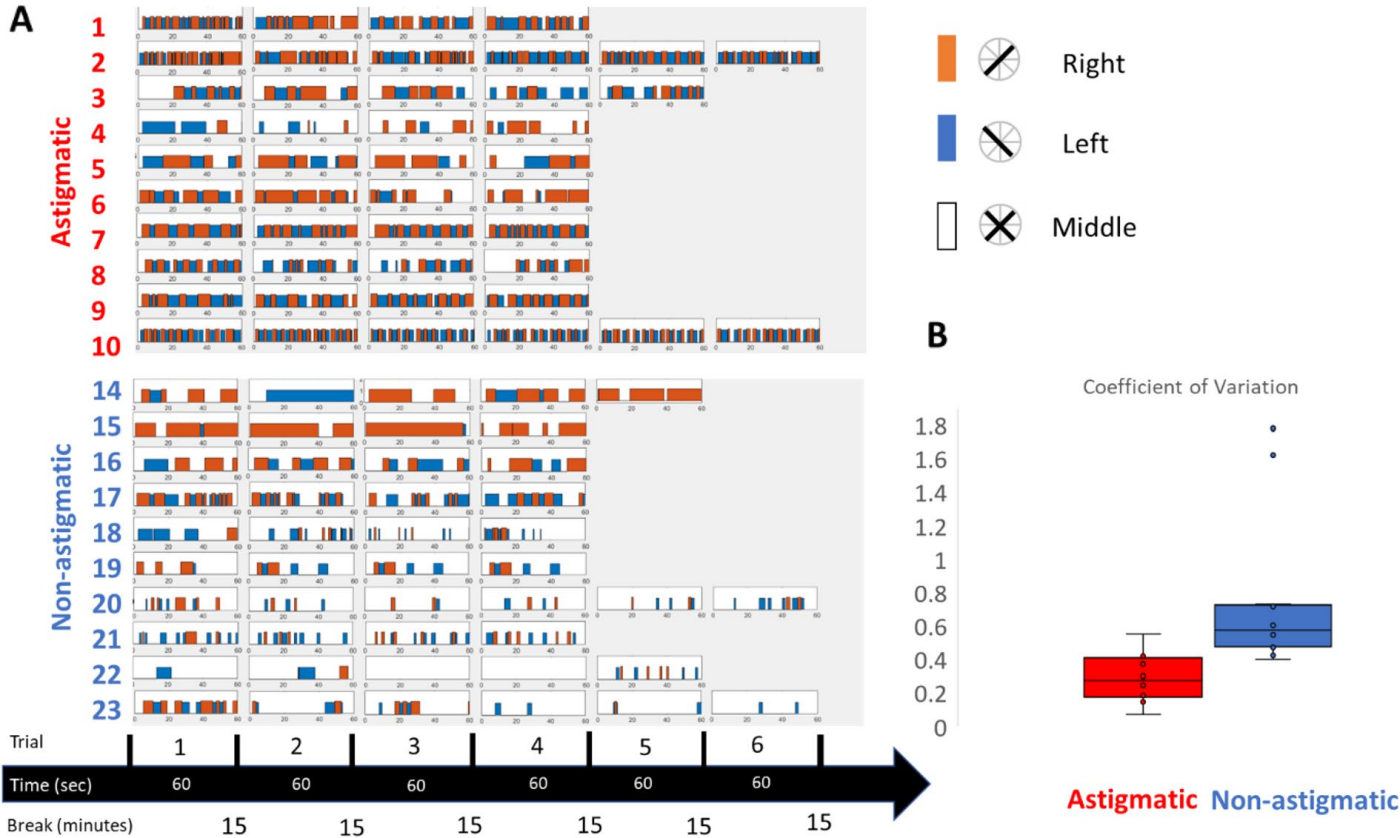
(**A**) Distribution of dominance and fusion duration between study groups for 100% contrast stimuli. The orange bar represents the right button, when the oblique line is at 2–8 o’clock, and the blue bar represents the opposite line (the 4–10 o’clock line); then the participants had to click on the left button. The participants were instructed to click on the middle button (see Fig. 6A). An equivalent perception of the black lines determines the fusion period between both eyes, which can vary between participants. (**B**) Coefficient of variation of the study group for 100% contrast. The box plot represents the interquartile range (IQR), which contains the middle 50% of the data. The horizontal line within the box represents the median, and the "whiskers" extend to the most extreme data points that are not considered outliers. Outliers are plotted as individual points outside of the whiskers. Each circle indicates one participant. The data were analyzed using a custom-designed MATLAB software, PSYVIEW, developed by Yoram Bonneh.

Figure 1A shows a significantly higher monocular alternation for oblique astigmatic participants compared with the non-astigmatic group for contrast stimuli of 100%. We defined one monocular alternation as one cycle of alternation when one orange bar is followed by one blue bar and vice versa. The oblique astigmatic group (OA) was characterized by the highest rate of monocular alternation and a low period of fusion, whereas the non-astigmatic group was characterized by a low rate of alternations.

We also calculated the study groups’ coefficient of variation of the monocular alternations^18^. The coefficient of variation is defined as the ratio between the standard deviation and the mean of each participant. Figure 1B shows an example of the different average coefficients of variation between the groups for a contrast of 100%. Here the coefficient of variation of the astigmatic group is significantly lower (*p* = 0.01363, Welch two-samples t-test, 0.29 ± 0.05; Mean ± SE; Median = 0.27; Minimum = 0.07; Maximum = 0.55) than that of the non-astigmatic group (0.78 ± 0.16, Mean ± SE; Median = 0.57; Minimum = 0.40; Maximum = 1.78), showing a clear difference of monocular alternation between the study groups. Accordingly, it was suggested^18^ that the alternation of the OA is most likely due to adaptation, whereas the alterations for the control group are more likely due to noise. The results for all tested contrasts of experiment 1 show the significant effect of group (two-way ANOVA, F(1,17.5) = 6.9 *p* = 0.0173, ${\eta }_{p}^{2}$=0.2822) and no interactions between the study groups and contrast, as well as no effect of contrast (two-way ANOVA, F(1.52.6) = 6.9 *p* = 0.37). The average coefficient of variation for 75% contrast for OA was 0.29 ± 0.05; 50% contrast: 0.40 ± 0.08; 25% contrast: 0.33 ± 0.05. For the control group: 75% contrast: 0.88 ± 0.28; 50% contrast: 0.81 ± 0.20; 25% contrast: 1.66 ± 0.95.

**Figure 2:**
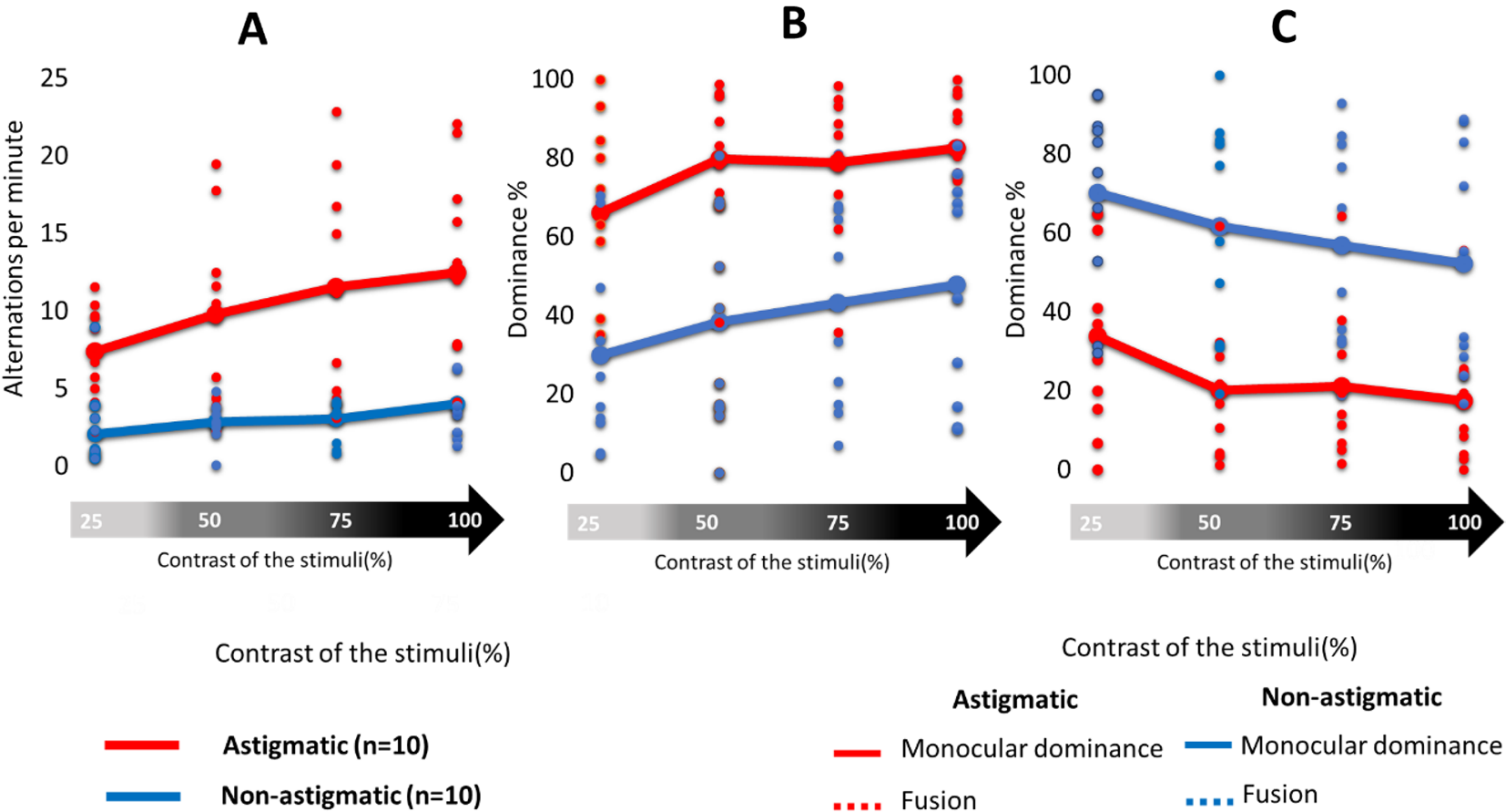
(**A**) Alternation between the eyes per minute according to contrast variation. Each dot represents the average of alternations per minute for each participant in both study groups (astigmatic and non-astigmatic). (**B**) Monocular dominance according to stimulus contrast variation. (**C**) Fusion dominance according to stimulus contrast variation. The red and blue bars denote the average between participants in each study group. Each dot indicates one participant.

Figure 2A as shown, there is a clear tendency for the astigmatic participants (denoted by a red line) to increase the number of alternations between their eyes in comparison with the non-astigmatic participants (denoted by a blue line). The results indicate that the astigmatic participants have a higher alternation rate than the control participants and that the rivalry increased significantly with increasing contrast, consistent with the literature^2^. However, the control group was weakly affected by contrast stimulus variation significantly less than the astigmatic group. A two-way mixed ANOVA was performed to determine the effect of contrast and group (astigmatic and non-astigmatic participants) on the number of alternations per minute. There was a significant effect of group (F(1,18) = 19.4005, *p* = 0.0003, ${\eta }_{p}^{2}$=0.2941) and a significant effect of contrast (F(1,18) = 19.4005, *p* = 0.0003, ${\eta }_{p}^{2}$ =0.5187) but no significant interaction between them (F(3,54) = 1.8929, *p* = 0.1417, ${\eta }_{p}^{2}$ =0.0952). The post hoc analysis showed that there were significant differences between astigmatic and non-astigmatic participants at all contrasts (*p* < 0.05 for all comparisons) as well as significant differences in astigmatic participants between contrasts 25 and 75 (*p* = 0.0069) and 25 and 100 (*p* = 0.0007).

Results shows that monocular dominance (Fig. 2B) and fusion (Fig. 2C) are affected by contrast stimulus variation. We calculated the monocular dominance using Eq. (1) (see the Methods section) and fusion dominance using Eq. (2) (see the Methods section). The astigmatic (the red bars) group shows a robust high tendency for monocular dominance, compared with the sphere group (the blue bars). These results are the direct consequence of the ability of the astigmatic participants to make more monocular alternations. As expected, we observed that both groups are affected by the variation in the contrast stimuli, indicating that the monocular dominance increases with contrast stimuli and that fusion decreases. A three-way mixed ANOVA was performed to test the effect of group (astigmatic and non-astigmatic participants) and the mode of dominance (monocular and fusion) on contrast variation. We found significant differences between fusion and monocular dominance (F (1,144) = 19.80; *p* = 0.0001; ${\eta }_{p}^{2}$ =0.1209). We also found a significant interaction between contrast and the mode of dominance (fusion and monocular), (F(3,144) = 3.869, *p* = 0.0107, ${\eta }_{p}^{2}$ =0.0746) as well as between groups (astigmatic and non-astigmatic) and the mode of dominance (F(1,144) = 98.7202; *p* = 0.0001, ${\eta }_{p}^{2}$ =0.4067). The post hoc analysis shows that there were significant differences between astigmatic and non-astigmatic participants at all contrasts (*p* < 0.01 for all comparisons). as well as significant differences in astigmatic participants between contrasts 25 and 75 (*p* = 0.0069, Cohen's d = 1.6913) and 25 and 100 (*p* = 0.0007, Cohen's d = 2.0692).

To minimize the impact of outliers on the analysis of the effects of group and contrast, a two-way mixed ANOVA was conducted under two conditions. In the first condition, "one" value of alternation per minute was excluded at a time, whereas in the second condition, "one" participant was excluded at a time. The statistical analyses revealed that the tests remained robust and significant between the groups despite the presence of outliers. Specifically, when "one" value of alternation per minute was excluded at a time, the group effect remained significantly robust, with a *p* value ranging from 0.0002 to 0.0005. Similarly, when "one" participant was excluded at a time, the group effect remained significant with a *p* value ranging from 0.00005 to 0.0008. Additionally, the effect of contrast was sustained when "one" participant was excluded at a time, with a *p* value ranging from 0.00004 to 0.001. Similarly, the effect of contrast variation was not strongly influenced by outliers and remained significant when "one" value of alternation per minute was excluded at a time. The *p* value varied between 0.00007 and 0.0007.

### Experiment 2

**Figure 3:**
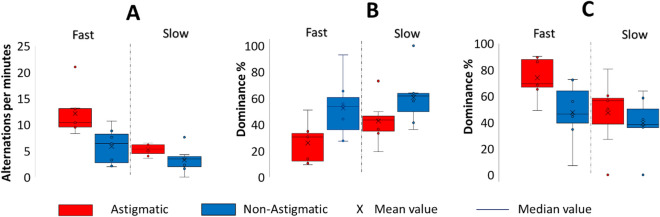
(**A**) The number of alternations per minute in both study groups for experiment 2. (**B**) Fusion dominance. (**C**) Monocular dominance between groups. The box plot represents the interquartile range (IQR), which contains the middle 50% of the data. The horizontal line within the box represents the median, and the "whiskers" extend to the most extreme data points that are not considered outliers. Outliers are plotted as individual points outside of the whiskers. Each circle denotes one participant.

In our previous experiment, we tested BR for the static condition. It was suggested that motion in a background can increase binocular rivalry. One possible explanation for this effect is that motion in the background can make it more difficult for the brain to integrate two separate images into a single percept^19,20^. Instead, the brain may focus more on the moving background, which can lead to stronger competition between the two images and greater fluctuations in perceptual dominance. Here we wanted to test BR under optic flow background speed variation. Figure 3A shows how an optic flow background affects BR. Importantly, we found that the speed of the background motion significantly affects the alternation rate per minute (two-way mixed ANOVA, F (1,12) = 34.12, *p* = 0.0001, ${\eta }_{p}^{2}$ =0.7398), and causes a significant effect between groups (two-way ANOVA, F(1,12) = 8.439, *p* = 0.0132, ${\eta }_{p}^{2}$ =0.4129. A significant interaction was observed between groups and flow background motion speed (two-way ANOVA, F(1,12) = 7.06, *p* = 0.0209, ${\eta }_{p}^{2}$ =0.3705).

The post hoc analysis shows that the faster flow significantly increases the number of alternations between eyes for the astigmatic group (*p* = 0.0002, Cohen's d = 3.21) but not appreciably for the non-astigmatic (*p* = 0.0585, Cohen's d = 1.2036). A different effect of speed was observed between the groups: the astigmatic group had a significantly higher number (*p* = 0.0022, Cohen's d = 2.94) of monocular alternations for the fast condition (mean ± SE; 12.13 ± 1.92; Median = 10.40; Minimum = 8.25; Maximum = 21) than the slow condition (mean ± SE; 5.25 ± 0.38; Median = 5.4; Minimum = 3.63; Maximum = 8.25). The control group seems to also be affected by the speed; for the fast condition, the number of alternations was higher) for the fast condition (Mean ± SE; 5.85 ± 1.40; Median = 6.38; Minimum = 2.00; Maximum = 10.68) than for the slow condition (mean ± SE; 3.28 ± 0.97; Median = 3.50; Minimum = 0.00; Maximum = 7.63); however, this difference was not significant (*p* = 0.0585). The main interesting result is the different rates between the study groups.

Figure 3B,C show how speed affects fusion and monocular dominance. We calculated the fusion dominance by using Eq. (2) (see the “Methods” section) and monocular dominance by using Eq. (1) (see the “Methods” section). A three-way mixed ANOVA was performed to test the effect of group (astigmatic and non-astigmatic participants) and the mode of dominance (monocular and fusion) on flow background motion speed. In this condition we observed a significant interaction between the study group and the mode of dominance (three-way mixed ANOVA, F(1,48) = 13.07, *p* = 0.0007, ${\eta }_{p}^{2}$ =0.214) and between motion speed and the mode of dominance (three-way ANOVA, F(1,48) = 7.54, *p* = 0.0084, ${\eta }_{p}^{2}$ =0.1358). The results in Fig. 3B indicate that the percentage of fusion dominance is higher in the fast condition for the non-astigmatic group than for the astigmatic group but that it is not significant (post hoc, *p* = 0.0582). Similarly, in Fig. 3C, the monocular dominance rate is higher (*p* = 0.01) for the astigmatic group but not statistically significant. For the slow condition (Fig. 3B,C) we did not observe a significant difference in fusion (*p* = 0.21) and monocular dominance (*p* = 0.56) between groups.

### Experiment 3

**Figure 4:**
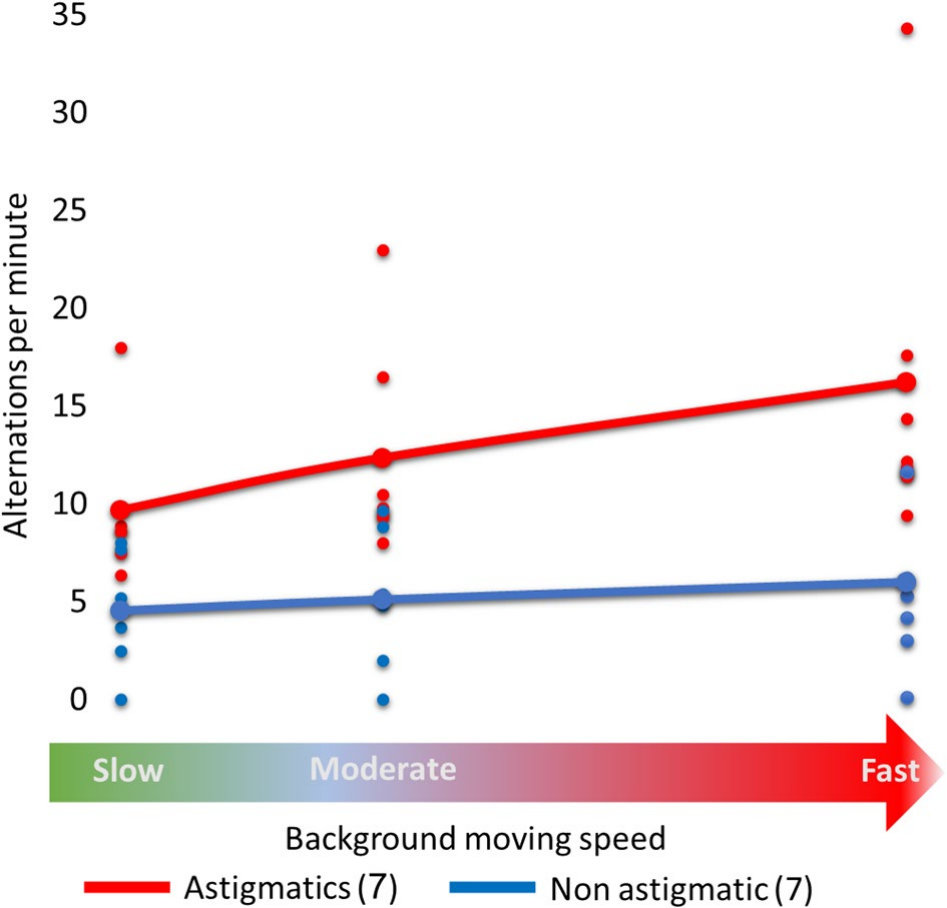
Alternations between eyes per minute according to the background moving speed (experience 3). Each dot represents the average alternation per minute for each participant in both study groups (astigmatic and non-astigmatic). The red and blue lines denote the average alternation between the participants in each study group. Each dot indicates one participant.

Our results in experiment 2 show how the speed variation background affects the binocular rivalry. Here we used the same static stimuli as in experiment 2, along with a background representing real-world motion using three different speed variations, as is described in the Methods section. Our real-world background consists of multiple different stimuli and interferences that could create a monocular bottleneck of information by allowing our brain to fuse two different images, and it increases monocular competition between the eyes. Figure 4 shows how the number of alternations per minute is affected by increasing the background motion speed under real-world conditions. Similar to the results of experiment 2, we observed that increasing the background moving speed using a video background increases the alternations per minute (two-way mixed ANOVA, F (2,24) = 10.57, *p* = 0.0005, ${\eta }_{p}^{2}$ =0.4684), and causes a significant group effect, especially the astigmatic group (two-way mixed ANOVA, F (1,12) = 8.49, *p* = 0.013, ${\eta }_{p}^{2}$ =0.4144). Another important result is the difference between the number of alternations between the study groups. Here, and similarly to the results of experiment 2, there is a clear tendency for astigmatic participants to make more alternations per minute compared with non-astigmatic participants for the three different speed levels. For the slow background speed, the astigmatic group made more alternations per minute than the non-astigmatism group (9.68 ± 1.55 vs. 4.5 ± 1.06, Mean ± SE; two-way mixed ANOVA, post hoc, *p* = 0.0106 Cohen's d = 4.4529). In the moderate background speed condition, the astigmatic group also made significantly more alternations (12.36 ± 2.62 vs. 5.12 ± 1.29, Mean ± SE; two-way mixed ANOVA, post hoc, *p* = 0.0392, Cohen’s d = 3.2665) than the non-astigmatic group. When the speed background was fast, the number of alternations per minute for astigmatic participants was much higher than for the control group, but it was not significant (16.23 ± 3.53 vs. 6.01 ± 1.63, Mean ± SE; two-way mixed ANOVA, post hoc, *p* = 0.1218). We also found a significant effect of speed in the astigmatic group (Fig. 4), Slow/moderate (*p* = 0.0392, Cohen's d =  − 1.59) and slow/fast (*p* = 0.0006, Cohen's d =  − 2.8006) and not for the non-astigmatic group (*p* = 0.56; *p* = 0.82).

## Discussion

Our results show a clear reduced ability to fuse images in participants with oblique astigmatism compared with the non-astigmatic ones when a blur is applied along the meridian of astigmatism. We also found, consistent with the literature, that BR could be affected by the stimulus contrast and by the speed and optic flow background speed variation.

### Competition for monocular dominance, a bottleneck of information

BR also results from interocular suppression between the monocular neurons of both eyes; this could be explained by monocular information or channel saturation, leading to increased competition between the eyes, which is affected by contrast variation and background optic flow motion speed. In the three experiments, the non-astigmatic group was less affected by contrast and the speed of motion background than was the oblique astigmatic group. We suggest that this observed disparity could result from a rapid monocular channel saturation combined with low interocular suppression between them.

### Continuous monocular competition

Wolfe’s model^7^ of binocular single vision suggests that the human visual system has evolved with two parallel pathways that extract the needed information from the input. This means that BR occurs unconsciously and belongs to the input selection process. According to our results, the OA participants show a reduced ability to fuse images in the three experiments when the blurred stimuli are overlapped with their optical blur; in other words, they fail to fuse images from both eyes when the monocular stimuli were blurred in an oblique direction but with a 90-degree difference between the eyes. We hypothesize that during the developmental period the OA participants were exposed to higher episodes of BR than were the non-OA participants; thus, they developed an increased sensitivity (reduced inter-ocular suppression) to BR^21^ during the critical period of development.

### Meridional rivalry regarding automatic blur compensation during development

One study^22^ showed that infants begin to become sensitive to BR by the age of 3 months. Other studies showed^23,24^ the link between OA and the increased risk of developing amblyopia in children and the specific difficulty needed to treat it^25^. Our recent study^16^ shows that lateral interactions continue to develop in children, maturing at the age of 5–6 years. In addition, it has been suggested that maturation of neural connectivity is experience dependent^26^, may result in meridional amblyopia^13^, and that adaptation to uncorrected visual input during development may be inferred from visual functions in adults^27^. Another study^15^ showed that adaptation to astigmatic blur is transferred to long-term memory that could be engaged when blur is reapplied or disengaged when blur is removed. Gu et al.^28^ suggest that neural adaptation after long-term exposure may play a key role in compensating for optical blur. Accordingly, during the developmental period, the oblique astigmatic participant is continuously exposed to mirror symmetry oblique blur, which may induce monocular competition that may increase the BR episodes. Therefore, we think that BR may result from the brain’s compensation for mirror symmetric blur that is transferred to long-term memory and then reapplied under the same blur condition in adult age.

### Natural binocular rivalry

It is important to emphasize that we measured rivalry under natural blur conditions. Most of the rivalry experiments are assessed by projecting two dissociated stimuli to eyes, consequently causing BR. Here, and for the first time, we show how natural blur, induced by OA and the natural shape of the eyes, could lead to BR. Apparently, this blur was a part of the long-term connectivity development that is still present in adults under specific conditions.

### The role of accommodation

Accommodation could play a role in the results. In our case, the pattern is robust enough to not require an accommodative effort. Astigmatic accommodation usually has a small amplitude (< 0.25 D) under monocular viewing conditions, and it is present only in some eyes^29,30^. The sitting distance (2 m) and adding a + 0.5D lens to each participant’s eyes neutralize the possibility to accommodate. In addition, each participant performed the experiment with + 1.00 D of myopic astigmatic blur, meaning that there is no possibility to accommodate under such conditions. Accommodation is activated when parallel light focuses behind the retina; here, under the blur condition, the parallel light focused before the retina, resulting in no accommodation activation. Here, we suggest that accommodation does not affect the obtained rivalry.

### The link to alternating strabismus

Alternative strabismus is perceptually characterized by alternative monocular suppression. We believe that binocular rivalry could be an intermediate step between diplopia and suppression. How could binocular rivalry be part of the development process that compensates for an ocular misalignment of an alternating strabismus? One study suggests that strabismic suppression might be achieved by the same mechanisms that cause binocular rivalry^31^. Other studies show that experience, which depends on abnormal conditions during the early period of development, could create long-term connectivities^26^ that could be reactivated under specific conditions in adult age^15^. Similarly, in our experiment it could be interesting to find traces that could reactivate the long-term connectivities generated during infancy, which may result from a perceptual compensation to alternating strabismus, such as improved binocular rivalry.

### Limitations of the study

We would like to emphasize that some factors, such as the speed of fluctuations in cortical alpha activity, could affect binocular rivalry^32^. A recent study^33^ correlates with the dynamics of perceptual alfa fluctuations, suggesting that inducing faster endogenous activity should accelerate perceptual switches. In addition, we asked the subjects to specify whether they wore glasses during their sensitive period; however, most of them were unable to provide accurate information.

Note that our study did not test BR under classic orthogonal grating stimuli. We think that the rate of alterations depends on the tested condition. We believe that OA participants would produce faster alternations than control participants would if they were tested with classic orthogonal grating stimuli. This possibility may support the effect found here and our speculation that OA develops during the critical period, leading to a decreased ability to fuse. This interesting option is planned to be tested in a future study.

### Conclusion

The results led us to speculate and suggest that in the context of an adjustment process, persistently distorted input exists during development; OA participants apparently experienced cycles of meridional binocular rivalry, and at an adult age, this ability was retained and reapplied under similar blur conditions. Therefore, this low ability to fuse could result from the abnormal development of neural connectivity during the critical period to compensate for the astigmatic blur.

## Methods

Twenty-six participants were enrolled in the study. They include a group of thirteen participants with oblique astigmatic error (n = 13) and a group of thirteen participants without astigmatic error, who served as the control group. The mean age for the OA group was 26.1 ± 2.73 (mean ± SD) and for the control group it was 25.4 ± 3.5 (mean ± SD): 8 men and 18 women; all were students at Bar-Ilan University. Twenty participants ran experiment 1, 14 participants ran experiment 2, and three participants ran one of them. The study protocol was approved by the Internal Review Board (IRB) of Bar-Ilan University. Informed consent was obtained from all participants and/or their legal guardian(s). All methods were performed in accordance with the relevant guidelines and regulations.

Each participant was included only after passing a full optometric eye exam, including visual acuity based on Snellen and logMar charts (ETDRS), autorefraction, retinoscopy, subjective, and binocular tests (Cover test), stereo vision (Random dot), Van Graef, fusional reserve, amplitude of accommodation, as well as negative and positive relative accommodation. As part of the clinical procedure of the optometric exam, the refractive power of each meridian (astigmatism) was determined and used for the experiment. Only participants with normal vision, a visual acuity of 6/6 (20/20) or better, no amblyopia, binocular disfunction, or ocular disease were enrolled.

### Measurement of astigmatism: the clock dial test for astigmatism

The clock dial test is a clinical method used by physicians^34^ to determine the astigmatic optical error. It helps to estimate the astigmatism’s power and orientation. It is based on the circular chart (an angular size of 250 arc minutes) with radii drawn at 30° intervals. The participant must first identify the sharpest line and then the physician must add cylindrical lenses of varying power oriented at a specific angle until all the radii appear equally clear.

**Figure 5:**
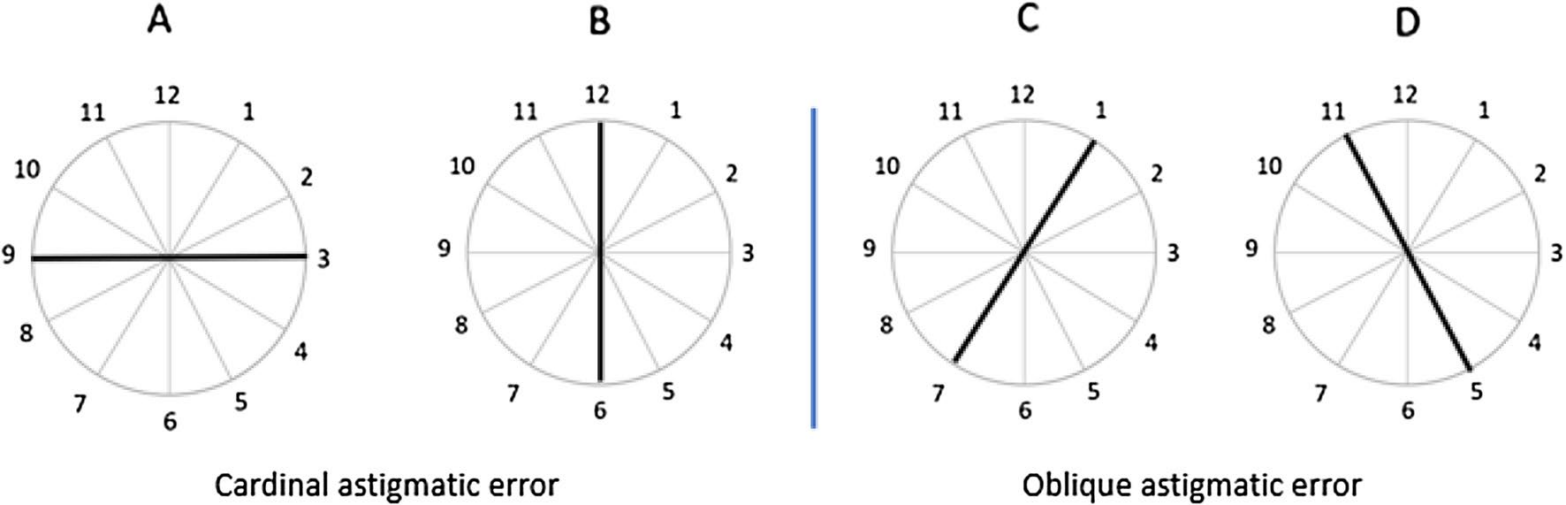
The clock dial test for astigmatism. (**A**) When the horizontal meridian is more curved than the vertical one. (**B**) When the vertical meridian is more curved than the horizontal one. (**C**) When the curved meridian is at an oblique orientation (30°). (**D**) When the curved meridian is at 150°.

As shown in Fig. 5, if the horizontal corneal meridian is more curved than the vertical one, the stimulus point will be perceived as a horizontal ellipse. Consequently, participants will perceive a more salient (black) horizontal line and vice versa. Figure 5 shows how participants with different astigmatic types perceive the clock. If we consider a compound myopic astigmatic participant, the salient line will represent the orientation of the blurriest meridian (Fig. 5A–D). In this test, the most prominent line refers to the uncorrected meridian orientation farthest from the retina, e.g., if the 1–7 o’clock line (see Fig. 5C) is perceived as more salient than the 5–11 o’clock line, the axis of astigmatism is approximately 135 degrees and vice versa. In clinical practice, this task is usually performed monocularly to test the power and orientation of the participants’ astigmatic meridian.

### Apparatus for the 3 experiments

All experiments were controlled using a PC, and the stimuli were displayed on a BENQ XL 2411 color monitor (24″) using custom software PSY developed by Bonneh et al.^35^. The mean display luminance was 40 cd/m^2^, in a dark environment. Screen resolution was 1920 × 1080; it subtended a visual angle of 14° × 8°. Stimuli were viewed from 200 cm. For experiments 1 and 2 the stimuli were displayed as gray-level modulation.

### Visual tasks

**Figure 6:**
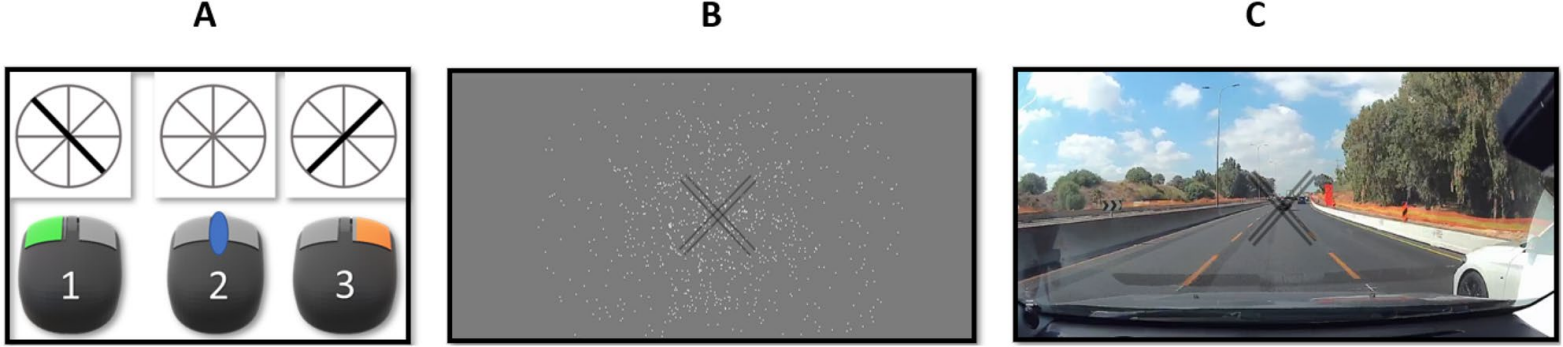
(**A**) Experiment 1—Illustration of the BR effect perceived by a non-corrected oblique astigmatic participant with 4 different contrasts: 25%, 50%, 75%, and 100%. Left monocular dominance. 2—Period of fusion (no rivalry). 3—Right monocular dominance. (**B**) Experiment 2—The optic flow experiments. (**C**) Experiment 3—The video background experiment.

For the 3 experiments, we measured the number of monocular alternations in each group when a blur of 1 diopter cylinder is applied to each eye, according to a meridian of astigmatism for the OA group. For the non-astigmatic participants, we induced blur using 1 diopter cylinder to each eye at 135° and 45°, similarly to the OA participants. We asked the participants to focus on the stimuli (see Fig. 6A) presented on the screen for 1 min. To minimize the accommodation effect for a seating distance of 2 m, each participant was binocularly blurred with a + 0.50 Diopter lens in addition to the + 1.00 Diopter cylinders adjusted to the participant’s axis to create an oblique blur effect. The task consisted of presentation for 1 min; each participant was asked to choose which meridian is the most prominent or that has an equivalent clarity and answer by pressing the mouse key (R, L, or none), according to the clock dial clock test. If the 2–8 o’clock oblique line was more pronounced, the participants had to click on the right button until they perceived the opposite line (the 4–10 o’clock line) as more salient; then they had to click on the left button. If the lines were perceived as similar, the participants were instructed to click on the middle button (see Fig. 6A). An equivalent perception of the black lines determines the fusion period between both eyes, which can vary between participants. For each experiment, the participants repeated the task from 4 to 6 times, and in order to avoid the effects of adaptation, they were asked to take up to a 15-min break between each repetition. Before each experiment we induced a blur of 1.00D and ensured that the stimuli were perceived similarly for both eyes of each participant and for all observers. The participants started running the experiment only after confirming that they saw the lines equally after covering each eye. It is important to emphasize that we induced a 1.00 D cylinder to both study groups, resulting in reduced sensitivity for both groups. The blurring conditions were the same for both study groups.

### The stimuli

#### Experiment 1

We chose stimuli based on the clock dial test (see Figs. 5, 6A); it includes 4 lines, disposed at a 45° angle from each other and surrounded by a circle, occupying a 5° angle. The stimuli were tested at 4 levels of contrast: 25%, 50%, 75%, and 100%. Each line width is 2.1 mm.

#### Experiment 2

We used the stimuli of two double static gray lines (see Fig. 6B): a 71 mm length and a 1.3 mm width, 5.3 mm from each other. Each dot size was 1.3 mm. The paired lines were disposed perpendicular to each other. We used an optic dot flow background when the dots moved from the center to the surround at 2 different speeds, slow and fast: slow when the dots moved at a speed of 5.3 mm/sec and fast when the dots moved at a speed of 26 mm/s.

#### Experiment 3

We investigated how BR could be affected by background motion speed variation in a real-world background situation consisting of multiple object stimuli, with different colors. Therefore, we used static stimuli similar to those in experiment 2 (see Fig. 6C); the background was video recorded with a NEXAR camera 2.0 at a constant driving speed (CDS) of 80 km/h for 1 min. We used Windows media player software to simulate 3 different speed variations: Slow = 1/2 CDS, moderate = CDS, and Fast = 2xCDS. Each participant repeated randomly the 3-speed background condition from 4 to 6 times with 15 min between each repetition.

### Data analysis

The data were analyzed using a custom-designed MATLAB software, PSYVIEW, developed by Yoram Bonneh. Two accumulated mouse clicks are presented in Fig. 1A; a right click (orange bar) and a left click (blue bar) are counted as one alternation. A period of fusion was considered as white space, when the participants equally perceived the lines and did not perform alternations. They were instructed to click continuously on the middle button of the mouse. In addition, we considered monocular dominance as the sum of the dominance time duration of both eyes (in s).

We calculated the percentage of fusion and monocular dominance:

1 $$ Monocular\;dominance\left( {\text{\% }} \right) = \frac{{Time\;of\;monocular\;dominance\left( {{\text{sec}}} \right)}}{{Presentation\;time\left( {{\text{sec}}} \right){ }}}{*}100 $$

2 $$ Fusion\left( {\text{\% }} \right) = \frac{{Time\;offusion\left( {{\text{sec}}} \right)}}{{Presentation\;time\left( {{\text{sec}}} \right)}}{*}100 $$

### Statistical analysis

Two- and three-way mixed ANOVA were performed to test the effect of 2 or 3 nominal variables (group, contrast, velocity, or mode) on continuous outcomes. Specifically, linear mixed-effect models were performed, and ANOVA was performed on the resulting models. All nominal variables were defined as fixed effects, and participant ID was defined as a random effect. Group (astigmatic and non-astigmatic) was defined as a between-participant factor, and contrast, velocity, and mode were defined as within-participant factors. Post hoc analysis was performed as pairwise comparisons defined by linear contrasts, and Benjamini–Hochberg (FDR) correction was applied to control for multiple testing. The normality of residuals and the homogeneity of variance assumptions were assessed graphically with diagnostic plots. *P* values less than 0.05 were considered statistically significant.

All analyses were conducted in the R statistical environment (R Core Team (2021). The partial Eta-squared $\left({\eta }_{p}^{2} \right)$ effect size was calculated for each effect and interaction term as follows:${\eta }_{p}^{2}=\frac{{SS}_{effect}}{{SS}_{effect}+{SS}_{error}}$, where SS is the sum of squares. In addition, Cohen's d effect sizes of pairwise comparisons were also calculated.

## Data Availability

The datasets used and/or analyzed during the current study are available from the corresponding author.
